# Supplementary material for: Content-rich biological network constructed by mining PubMed abstracts
Source: BMC Bioinformatics. 2004 Oct 8;5:147. doi: 10.1186/1471-2105-5-147 (PMC528731; doi:10.1186/1471-2105-5-147)
Supplement: Additional File 2 — The original results of the above study (non-essential files are deleted to keep the file size under the limit set by BMC bioinformatics). [file 1471-2105-5-147-S2.bz2 › chilibotAdditionalFile2/dip05/244ID8921390E800/html/TP53_UBE2I.html]

 


 **TP53** and **UBE2I** 
  
Found 2 abstracts in PubMed, retrieved 2.  
 

 What does Google say? 
 PDF only 
| .edu only 

---

**Interactive relationship** (e.g. stimulation, inhibition, etc)

**Inhibitory relationship**- Analysis of the enzymatic cascade involved in the conjugation process reveals that the ubiquitin carrier protein  [ **UBE2I** ]  E2 F1 and its human homolog UbcH5, which target the tumor suppressor p53  [ **TP53** ]  for degradation, are also involved in c Fos recognition.  Ref: 8524278 Mol Cell Biol, 1995
**Neutral relationship**
